# 

**Published:** 1883-02-20

**Authors:** 


### TABLE OF CONTENTS.

ORIGINAL AND SELECTED ARTICLES.

Clinical Remarks on Dyspepsia, by R. C. Word, M. D., Professor of Physiology in the Southern Medical College, Atlanta, Ga., 41. A case of Difficult Labor, by A. W. Reese, M. D, 49. Two cases of Tracheotomy, by C. G. Jennings, M. D., Detroit, 53. Some Points on the Reduction of Hernia, by J. S. Wight, M. D., 56.

ABSTRACTS AND GLEANINGS.

Bromide of Ethyl, the Most Perfect Anæsthetic for Short, Painful Surgical Operations, 58. The Acceleration of Delivery in Peurperal Convulsions, 63. Hemorrhagic Malerial fever, 65. Physiological and Therapeutical Effects of Quassine, 68. Aunt Betsy Simpson's case, 69. A New point in the Diagnosis of Femoral Luxations; Celerina, 70.

SCIENTIFIC ITEMS.

Burdensome Inventions; Progress of Electrical Lighting; The Signal Service, 71. Electricity in the Parlor, etc.; Signaling by Electricity, 72

PRACTICAL NOTES AND FORMULÆ.

New Treatment for Vaginitis; A Valuable Mixture for Chills; Bromides and Chloral in Whooping Cough: To Correct the Odor of the Lochia after Child-Birth, 73. Disguising the Taste of Quinine; Asthma Mixture; Pruritus Vulvæ; Hair Wash, 74.—Tasteless Quinine; Treatment of Pertussis; To Harden Nipples, 75.

EDITORIALS AND MISCELLANEOUS.

Editorial Notices, 76. U. S. Dispensatory; The N. Y. Code of Ethics; Marriage of Prof. Powell, 77. Medical Association of Missiouri; Book Notices, 78. Pamphlets Received; Small-pox; Prevailing Diseases, 79. Receipted; Special Notices, 80.

